# The Longitudinal Relationship Between Allostatic Load and Multimorbidity Among Older Americans

**DOI:** 10.3390/geriatrics10040084

**Published:** 2025-06-26

**Authors:** Rolla Mira, Jonathon Timothy Newton, Wael Sabbah

**Affiliations:** Faculty of Dentistry, Oral & Craniofacial Sciences, King’s College London, Bessemer Road, Denmark Hill, London SE5 9RS, UK; rolla.mira@kcl.ac.uk (R.M.); tim.newton@kcl.ac.uk (J.T.N.)

**Keywords:** ageing, allostasis, longitudinal studies, multimorbidity

## Abstract

**Background:** To examine the association between allostatic load and the progression of multimorbidity and the role of socioeconomic factors among older Americans. **Methods:** Data from the Health and Retirement Study (HRS), a longitudinal study of older American adults, were used. Data were included from waves 8 (2006), 10 (2010), 11 (2012), and 13 (2016). Self-reported diagnoses of five chronic conditions (diabetes, heart disease, lung diseases, cancer, and stroke) indicated multimorbidity and were dichotomised to reflect having two or more conditions versus one or fewer. Multimorbidity in 2006 was subtracted from that in 2016 to calculate ten-year change in multimorbidity. Sociodemographic data (age, gender, education, and wealth) were from wave 8 (2006). Behaviours (physical activity and smoking) were from wave 10 (2010). Allostatic load, indicated by five biomarkers (waist circumference, high blood pressure, glycosylated haemoglobin, high-density lipoprotein, and c-reactive protein), was from wave 11 (2012). Structural Equation Modelling (SEM) was used to assess the longitudinal association between the aforementioned factors and the incidence of multimorbidity in 2016. Results: Given that allostatic load was assessed in a subsample of HRS, 8222 were excluded for lack of relevant data. A total of 3336 participants were included in the final analysis. The incidence of multimorbidity in 2016 was 19%. Allostatic load in 2012 was significantly associated with the incidence of multimorbidity in 2016 (estimate 0.10, 95% Confidence Interval (CI) 0.07, 0.14); in other words, for an additional marker of allostatic load, there was an average 0.1 change in the incidence of multimorbidity. Wealth and education (2006) were indirectly associated with multimorbidity through allostatic load and behaviours. Smoking (2010) was positively associated with multimorbidity in 2016, while physical activity showed a negative association. **Conclusions:** Biological markers of stress indicated by allostatic load were associated with multimorbidity. Adverse socioeconomic conditions appear to induce allostatic load and risk behaviours, which impact the progression of multimorbidity.

## 1. Background

Interest in multimorbidity has grown over the past several years due to its significant impact on patients and their families, as well as on healthcare systems and society [1,2,3]. Multimorbidity is defined as the presence of at least two chronic conditions in the same individual [4]. Patients with multiple chronic conditions frequently experience worse health outcomes, such as frailty, a decline in physical and mental health functioning, and higher mortality rates [5,6,7]. In the United States, several serious, life-threatening, and prevalent chronic conditions, such as cardiovascular diseases, diabetes, cancer, stroke and chronic pulmonary diseases, are highlighted in research and policies on multimorbidity for their impact on morbidity, mortality, and the healthcare system [8]. The frequency of multimorbidity is significantly influenced by unfavourable socioeconomic circumstances [9]. These include early and adult life socioeconomic position (SEP), deprivation level, education, and income [10]. Furthermore, according to two studies conducted in the USA, multimorbidity rates were greater among ethnic minorities, particularly among Black and Hispanic Americans [11,12]. On the other hand, chronic stressors are also considered to be a serious health hazard in the twenty-first century. They are linked to chronic noncommunicable diseases and health risk behaviours [13]. Several studies examined the association between allostatic load (AL), as a marker of chronic stress, and chronic conditions [14,15,16]. BS McEwen and E Stellar [17] first used the term “allostatic load” in 1993. It was defined by the cumulative impact of experiences in daily life that include both minor challenges (subtle and long-standing life situations) and major challenges (life events), as well as the physiological effects of the resulting health-damaging behaviours, such as poor sleep and circadian disruption, lack of exercise, smoking, and unhealthy diet [17,18,19]. Allostatic load is the physiological wear and tear that the body sustains throughout the life course [18,20,21]. Therefore, AL is identified as a marker of stress, as it reflects repeated exposure to stressful life events and the individual’s ability to cope with these events [22,23].

Biomarkers of AL have been the subject of several studies [19,24,25]. They include cortisol, dehydroepiandrosterone (DHEA), epinephrine, and norepinephrine, which are considered primary mediators of AL due to their direct link with adrenal function. Secondary mediators include biomarkers such as cholesterol, glycosylated haemoglobin, resting systolic and diastolic blood pressure, c-reactive protein, and waist/hip ratio [23,25]. The tertiary mediators are the biological markers of diseases resulting from a condition of allostatic load [26].

Allostatic load can result from several different circumstances, including (a) exposure to frequent stressors that may determine a status of chronic stress and repeated physiological arousal; (b) lack of adaptation to repeated stressors; (c) inability to shut off the stress response after a stressor is terminated; and (d) insufficient response to deal with the stressor [18,27].

The biomarkers of allostatic load are considered risk factors for several chronic conditions such as cardiovascular diseases, stroke, and diabetes [14,23]. Often, these stressors result from adverse socioeconomic conditions [25,28]. It is, therefore, reasonable to hypothesise that allostatic load will be linked to both adverse socioeconomic status on one hand and several chronic conditions on the other.

Despite the known association between AL and several individual chronic conditions [14,16,25], there is a lack of studies that examined the association of AL with the progression of multimorbidity. Most of the studies that assessed the relationship between allostatic load and chronic conditions focused on individual conditions such as cognitive decline, cardiovascular diseases, cancer, diabetes, [14,16], or used cross-sectional data [21], but no known studies assessed the relationship with a combination of chronic conditions reflecting multimorbidity. This paper aims to examine the relationship between allostatic load and the progress of multimorbidity over 10 years among older Americans and the role of socioeconomic factors in this relationship.

## 2. Materials and Methods

### 2.1. Study Design

This study used longitudinal data from the Health and Retirement Study (HRS), a nationally representative survey of older Americans, which collects data from participants in the 50 states. Since 1992, the HRS has collected data biennially with response rates consistently above 80%. Before each interview, participants received a confidentiality statement and gave their verbal agreement. Since 2006, half of the panel respondents have additionally undergone upgraded face-to-face interviews every two years. Since the HRS data are available in the public domain without identifying information about specific individuals, ethical approval was not required for the current analysis.

### 2.2. Study Sample

The study sample consists of older American adults drawn from the HRS, a national sample of Americans aged 50 and over [29]. Indeed, most longitudinal surveys of older adults recruit participants at age 50 to observe how their health and social circumstances change over the years as they age. To investigate allostatic load (biological markers of stress), we used data from the Sensitive Health Data Panel/Biomarker Data. Biomarker data was available from 2006 to 2016. This period corresponds to waves 8–13. We selected 3 biomarkers to use as indicators of allostatic load: high-density lipoprotein cholesterol (HDL), glycosylated haemoglobin (HbA1c), and C-reactive protein (CRP). In addition, we included two markers (waist circumference and blood pressure) from the RAND HRS Longitudinal File 2018 [30]. The selection of these biomarkers was based on their identification as secondary parameters of allostatic load [23,25]. It is worth noting that there are other direct markers of stress, such as cortisol, epinephrine, and norepinephrine, and other secondary markers that were not available in the database.

### 2.3. Study Variables

The analysis used demographic and socioeconomic data from 2006, behavioural data from 2010, allostatic load from 2012, and multimorbidity incidence in 2016 (accounting for multimorbidity in 2006). Demographic factors included age and gender (male, female). Socioeconomic indicators included education (less than high school, high school, some college, and college or above) and total wealth.

Wealth measures were reported in nominal dollars and calculated as the sum of the appropriate wealth components minus debts. It is worth noting that wealth is a proper indicator of socioeconomic conditions among older adults, as it reflects financial security. It was categorised into quartiles (highest, second highest, second lowest, and lowest). In this analysis, wealth was from wave 8 (2006).

Behavioural factors included physical activity and smoking status and were included from wave 10 (2010). Light, moderate, and vigorous physical activity were classified into three groups based on intensity. Light activities included dancing, gardening, golfing, bowling, and strolling. Moderate activities included mopping the floor, cleaning the car, or stretching exercises. Vigorous activities included aerobics, running, swimming, or bicycling. The following scores were given for each type of physical activity: Mild activity frequency was scored as 3+ times/week = 1, 1–2 times/week = 0.43, 1–2 times/month = 0.14, 1/month = 0.07, and never = 0. The number of moderate and vigorous daily activities was determined in the same way, then multiplied by 2 and 3, respectively. A scale of physical activities was calculated by adding the sums of the values for mild, moderate, and vigorous activities. This variable has been used in previous studies that used HRS [11].

Smoking was reported in HRS as current smokers, former smokers (those who smoked at least 100 cigarettes), and those who had never smoked. The variable was categorised to indicate never/former smoker versus current smoker. It is worth noting that there are several other variables included in HRS, such as variables on several medications and markers of psychological well-being, but they were not included in the analysis, as their inclusion would lead to over-adjustment. Furthermore, inclusion of some other variables, such as BMI and high blood pressure, would be redundant, as markers of allostatic load include indicators of these variables.

### 2.4. Indicators of Allostatic Load

Earlier studies on allostatic load included several secondary markers [17,18,23,25,26]. In the current analysis, we selected markers that were consistent with what is suggested in these earlier studies and were available in the Health and Retirement Study. High-density lipoprotein (HDL) was categorised as normal: <40 mg/dL, high: ≥40 mg/dL for men, and normal < 50 mg/dL, high ≥ 50 mg/dL for women [31]. Glycosylated haemoglobin (HbA1c) was recorded as a normal level < 6.5%, diabetic ≥ 6.5% (above 48 mmol/mol approx.) [32]. C-reactive protein (CRP) was recorded as normal < 3 mg/L, high ≥ 3 mg/L [33,34]. Waist circumference was categorised as normal ≤ 39 inches, high ≥ 40 inches for men, and normal ≤ 34 inches, high ≥ 35 inches for women [35]. High blood pressure was indicated by systolic ≥ 140 mm Hg and diastolic ≥ 90 mm Hg for both genders [36]. These five indicators were added to create an allostatic load variable ranging from 0 to 5. While there are other markers of allostatic load [37], these were the available markers in HRS consistent with the secondary parameters of allostatic load [23,25]. Allostatic load markers were from wave 11 (2012).

Multimorbidity was indicated by self-reported diagnoses of 5 chronic conditions: diabetes, heart conditions, lung diseases, cancer, and stroke. These diseases were chosen because they are the main causes of disability and mortality in the USA and are highly common among older adults [8]. A multimorbidity score ranging from 0 to 5 was created by adding these 5 conditions. Multimorbidity scores in 2006 and 2016 were dichotomised to indicate having 2 or more conditions versus one or fewer. Multimorbidity in 2006 was subtracted from that in 2016 to calculate the ten-year incidence of multimorbidity.

### 2.5. Statistical Analysis

The analysis included participants with complete data in all included variables in the included waves (2006, 2010, 2012, 2016). The percentage of participants with multimorbidity in 2016 (two or more conditions, excluding those with multimorbidity in 2006) was calculated. Mean allostatic load in 2012 and distribution of behaviours (2010) and socioeconomic factors (2006) were also calculated for those included in the final analysis.

Structural Equation Modelling (SEM) was used to assess the longitudinal association between socioeconomic factors (2006), behaviours (2010), allostatic load (2012), and incidence of multimorbidity in 2016. SEM tested the direct relationship between each of the socioeconomic factors and behaviours, and multimorbidity, as well as the indirect relationship through allostatic load. The standardised estimation method was used in SEM analysis. The Tucker–Lewis Index (TLI), the Comparative Fit Index (CFI), and the Root Mean Square Error of Approximation (RMSEA) were used to assess the goodness of fit.

For sensitivity analysis, a logistic regression model testing the relationship between allostatic load and incidence of multimorbidity, adjusting for all variables included in the SEM, was conducted.

## 3. Results

### 3.1. Characteristics of the Study Sample

**Figure 1:**
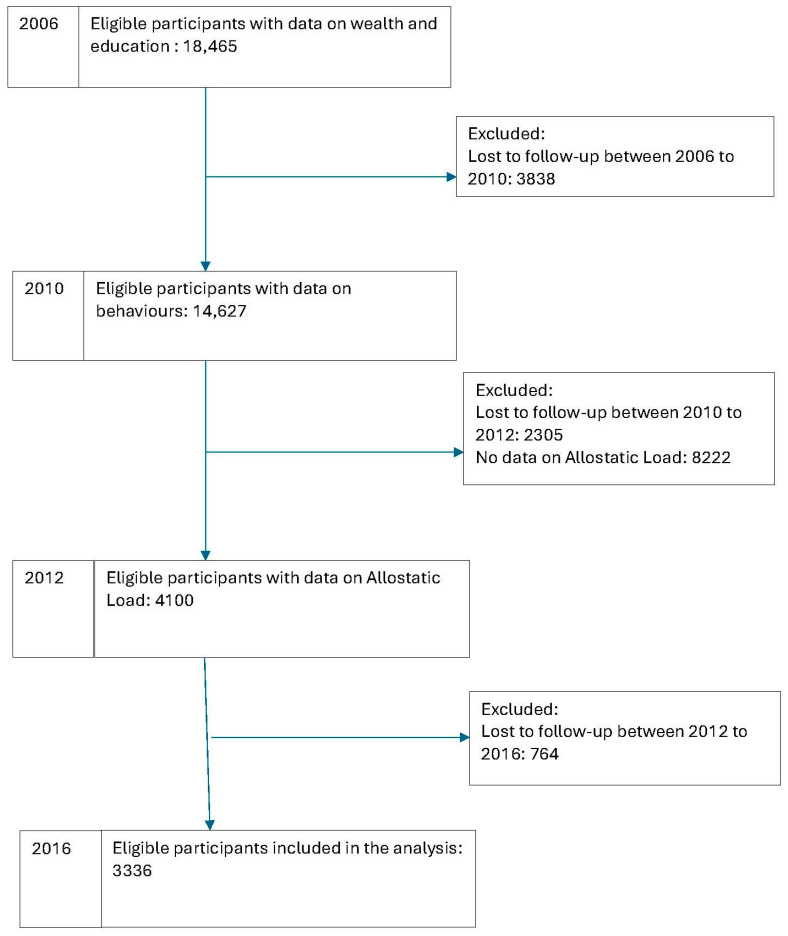
Flowchart of the number of participants included in the analysis from 2006 to 2016.

The analysis included 3336 participants with complete data on all included variables between 2006 and 2016. Given that biomarkers of allostatic load were collected from a subsample of the survey, 8222 participants were excluded. Most of the other excluded participants were missed at follow-up waves over 10 years (Figure 1).

Table 1 shows the distribution of all variables included in the analysis. The mean age in 2006 was 64.48. The 10-year incidence of multimorbidity in 2016 was 19%. The mean of allostatic load in 2012 was 1.77 (Table 1).

### 3.2. Association Between Allostatic Load and Incidence of Multimorbidity

**Figure 2:**
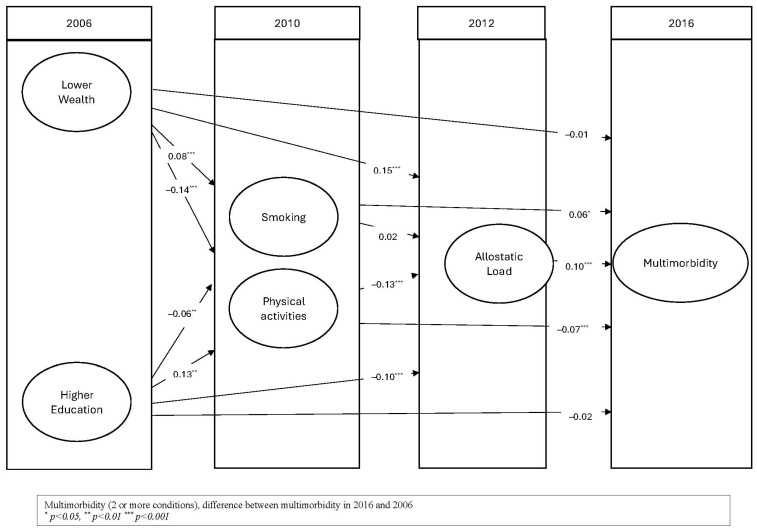
Pathways linking allostatic load to multimorbidity.

Table 2 shows the results from SEM. Allostatic load in 2012 was positively and significantly associated with multimorbidity in 2016 (estimate 0.10, 95% Confidence Interval (CI) 0.07, 0.14), indicating that for each additional marker of allostatic load, there is an average of 0.1 change in the incidence of multimorbidity. Smoking was positively associated with multimorbidity, while physical activities showed a negative association. The two behaviours also demonstrated an indirect association with multimorbidity through allostatic load. On the other hand, wealth and education were indirectly associated with multimorbidity through allostatic load, but they did not show a significant direct association with multimorbidity. The model showed proper fitting values. The direct and indirect associations with multimorbidity are depicted in Figure 2.

### 3.3. Sensitivity Analysis

In the sensitivity analysis, allostatic load was significantly associated with multimorbidity, with an odds ratio of 1.27 (95%CI: 1.17, 1.38) in a model adjusting for all variables included in the SEM.

## 4. Discussion

This study assessed the relationship between allostatic load and the progress of multimorbidity among older adults in the USA. The main findings of this study indicate that higher levels of allostatic load in 2012 were associated with a greater incidence of multimorbidity in 2016. This finding is consistent with previous studies showing that a higher level of allostatic load usually precedes the development of many chronic conditions [14,16,38]. It is important to note that the significant association between allostatic load and multimorbidity persisted after accounting for the direct relation between socioeconomic and behavioural factors with multimorbidity.

Adverse socioeconomic factors impact psychological well-being, leading to an increase in the levels of stressors. Repeated and chronic exposure to stressors leads to wear and tear in the body systems, which increases allostatic load, a risk factor for chronic conditions [23,25]. Unsurprisingly, earlier studies demonstrated that lower socioeconomic factors were associated with elevated levels of allostatic load [14,38]. The current analysis clearly showed that adverse socioeconomic conditions were associated with a greater level of allostatic load, and with health behaviours [11,39], which, in turn, were linked to multimorbidity. However, there was no direct relationship between indicators of socioeconomic factors at baseline in 2006 and the incidence of multimorbidity in 2016. The relationship appeared to be indirect through allostatic load and health risk behaviours. In other words, socioeconomic inequalities in multimorbidity were explained by their impact on stressors indicated by allostatic load and health behaviours. It is worth noting that earlier studies demonstrated socioeconomic inequalities in the progress of multimorbidity [10,11]. Ethnic/racial inequality is another potential confounder in the observed relationship; however, earlier studies demonstrated that ethnic/racial inequalities in multimorbidity were explained by socioeconomic factors [11].

Similarly to earlier studies [40,41], health-promoting behaviours were also inversely associated with higher levels of allostatic load and with multimorbidity. In other words, health behaviours demonstrated both direct and indirect relationships with multimorbidity through allostatic load.

It is worth noting that we used the most common life-threatening chronic conditions in the USA [8], and the allostatic load variable included most of the common secondary indicators of allostatic load available in the HRS [23,25].

The strengths of this study lie in using a large longitudinal dataset of older Americans to measure the association between allostatic load and the ten-year incidence of multimorbidity, accounting for behavioural and socioeconomic factors. While it could be argued that individual markers of allostatic load are highly correlated with individual chronic conditions, e.g., glycated haemoglobin and diabetes, the analysis assessed the relationship between the combined markers of allostatic load and a combined variable of multimorbidity. Furthermore, the time sequence of each of the main exposures, namely socioeconomic factors, behaviours, allostatic load, and incidence of multimorbidity in 2016, allows for a conclusion about temporality. In other words, in the current analysis, the exposure (allostatic load in 2012) preceded the outcome (multimorbidity incidence in 2016), indicating a temporal relationship. On the other hand, a causal relationship could be established through intervention studies. To the best of our knowledge, no known study used longitudinal data to assess the temporal relationship between allostatic load and multimorbidity. This research indicates that stress (allostatic load) brought on by unfavourable socioeconomic conditions increases the chance of developing multimorbidity. The findings highlight the importance of identifying and targeting those exposed to adverse socioeconomic conditions and chronic stressors to enable the development of health promotion interventions that encompass social, psychological, and behavioural factors to tackle multimorbidity among older adults. Furthermore, assessment of biomarkers of allostatic load should be routinely incorporated in assessments of older adults to identify those at higher risk of progression of multimorbidity and enable the development of appropriate interventions. The current analysis highlighted some behaviours linked to multimorbidity through allostatic load, such as smoking and physical activities. These behaviours, among others, should be included in interventions targeting older adults with higher levels of stress to reduce the impact on the biomarkers and on multimorbidity. For example, earlier studies argued that management of pain and home exercise could delay disease progression [42]. While the current study examined the relationship among allostatic load as a marker of chronic stress, adverse socioeconomic factors, and the progression of multimorbidity, other factors could contribute to allostatic load and the progression of multimorbidity, which were not examined here. For example, early-life socioeconomic factors could impact both allostatic load and multimorbidity [43,44]. Neighbourhood characteristics, area deprivation, social relationships and participation, and discrimination, among other factors, could also impact allostatic load and multimorbidity [28]. Future research on this area should incorporate geospatial and community-level data when available. There are a few limitations worth mentioning. First, although the analysis of the longitudinal data supports temporal association, it does not indicate causality. Second, the use of self-reported diagnoses of chronic conditions is less reliable than data from medical records, and it could possibly lead to misclassification of the participants and attenuate the association. However, this is not feasible in national surveys. Third, there are other markers of allostatic load that were not available in the database; however, we included five of the important markers. Finally, self-reported behaviours and socioeconomic factors could also be subjected to recall bias.

## 5. Conclusions

This study demonstrated an association between allostatic load as a marker of stress and the incidence of multimorbidity. Allostatic load explained the impact of adverse socioeconomic factors on multimorbidity. Practical implications of the findings include enabling risk stratification of those at higher risk of allostatic load and multimorbidity to inform public health policies. The results emphasise the significance of identifying those who have chronic stressors induced by adverse socioeconomic conditions to target them with effective health promotion interventions that take into consideration the environmental, financial, psychological, and behavioural determinants of health, as these interventions will help enable people to increase control over their own health and cover a wide range of social and environmental interventions designed to benefit and protect people’s health and quality of life by addressing and preventing the root causes of ill health, not just focusing on treatment and cure.

## Figures and Tables

**Table 1:** Characteristics of the study sample—Health and Retirement Study (USA) (2006–2016) (N:3336).

Year	Variables		Percentage/Mean
2006	Mean age		64.48 (95% CI: 64.19, 64.77)
	Sex	Males	38.9%
		Females	61.1%
	Education (2006)	<high school	22.5%
		High school	31.0%
		Some college	22.7%
		College or higher	23.8%
	Wealth (2006)	Highest	33.1%
		2nd highest	27.4%
		2nd lowest	25.2%
		lowest	14.3%
2010	Smoking	Former/never	89.3%
		current	10.7%
	Mean physical activities		1.37 (95% CI: 1.33, 1.41)
2012	Mean allostatic load markers		1.77 (95% CI: 1.74, 1.82)
2016	Multimorbidity (incidence between 2006–2016)		19.0%

**Table 2:** Path coefficient of factors associated with incidence of multimorbidity among older American adults—Health and Retirement Study (2006–2016) (N:3336).

Variables		Estimate (Coefficient) (95% CI)
Smoking (2010)		
	Lower wealth (2006)	0.08 *** (0.09, 0.16)
	Higher education (2006)	−0.05 ** (−0.09, −0.02)
Physical activities (2010)		
	Lower wealth (2006)	−0.14 *** (−0.18, −0.11)
	Higher education (2006)	0.13 *** (0.09, 0.17)
Allostatic load (2012)		
	Lower wealth (2006)	0.15 *** (0.11, 0.18)
	Higher education (2006)	−0.10 *** (−0.14, −0.07)
	Smoking (2010)	0.02 (−0.1, 0.05)
	Physical activities (2010)	−0.13 *** (−0.17, −0.09)
Multimorbidity ^±^ (2016)		
	Gender (Female)	−0.08 *** (−0.11, −0.05)
	Age (2006)	0.11 *** (0.07, 0.14)
	Lower wealth (2006)	−0.01 (−0.05, 0.03)
	Higher education (2006)	−0.02 (−0.06, 0.01)
	Smoking (2010)	0.06 ** (0.01, 0.08)
	Physical activities (2010)	−0.07 *** (−0.10, −0.04)
	Allostatic load	0.10 *** (0.07, 0.14)
Model fit		
	RMSEA	0.035 (0.021, 0.42)
	CFI	1.0
	TLI	1.0

^±^ Incidence of multimorbidity (2 or more conditions), difference between multimorbidity in 2016 and 2006. ** *p* < 0.01, *** *p* < 0.001.

## Data Availability

Data used in this research is available on https://hrs.isr.umich.edu/data-products (accessed on 15 April 2025).
